# Vesicovaginal fistula: An unusual complication of laparoscopic assisted nephroureterectomy

**DOI:** 10.4103/0972-9941.26648

**Published:** 2006-06

**Authors:** Rajiv G Pillai, Ameet S Patel, Anant Kumar

**Affiliations:** Departments of Urology, Addenbrookes' Hospital, Cambridge, UK

**Keywords:** Laparoscopic nephroureterectomy, operative complications, vesico-vaginal fistula, perative technique

## Abstract

We report a case of vesicovaginal fistula in 71-year-old lady who had previously undergone a lapascopic assisted nephroureterectomy for transitional cell carcinoma in her right ureter and kidney. The surgery was uncomplicated with no post-operative problems and was discharged on day on seven. She later presented five weeks following the initial operation with signs and symptoms suggestive of a vesicovaginal fistula, which was confirmed on cystogram and flexible cystosopy. She proceeded to have an abdominal (O'Connor's) repair of the fistula together with cystodiathermy for a few superficial bladder recurrences. The area of the fistula (within the bladder) was noted to be tumour free. She had an uneventful post-operative recovery and was discharged from hospital on day 11. At six month follow-up, there was another superficial recurrence in the bladder that was resected, with no sign of fistula.

## INTRODUCTION

Vesicovaginal fistula is a well noted complication following conventional and laparoscopic gynaecological surgery.[1,2] Here we report an unusual case following laparoscopic assisted nephroureterectomy. Review of the literature revealed just a single reported case of vesicovaginal fistula which arose postoperatively after removal of the ureter by intussuseption method for renal pelvic tumour.[3]

## CASE REPORT

A 71-year old obese lady underwent a laparoscopic assisted nephroureterectomy for a transitional cell carcinoma (pT3G2) of the right kidney and ureter. The lower end of the ureter was mobilised and excised using a small (7 cm) Gibson's incision and the same was also used to deliver the kidney. She also underwent cystoscopy and cystodiathermy of superficial bladder transitional cell carcinoma at the same time. She did well postoperatively and was discharged in a week following the initial operation.

**Figure 1:**
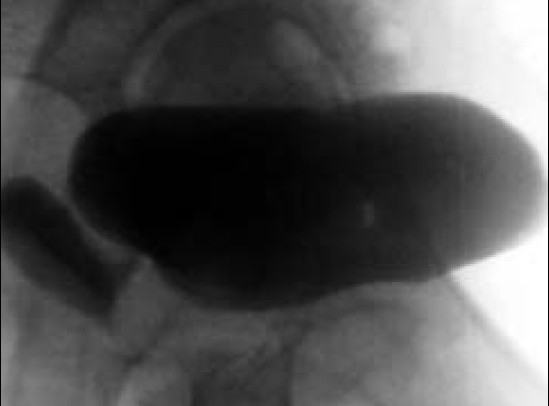
Cystogram showing vesicovaginal fistula

She developed a urinary leak five weeks following the operation and was found to have a vesicovaginal fistula, which was confirmed on cystogram [Figure 1] and flexible cystoscopy. She underwent an abdominal (O'Connor's) repair of the fistula five months following the initial operation. She also had cystodiathermy for some recurrent superficial bladder lesions at the same time and the area of fistula in the bladder was found free of any tumour. She did well postoperatively and was discharged 11 days after the operation. She has been seen in follow-up clinic after 6 months where there was another superficial recurrence, which was resected. Presently she is doing well.

## DISCUSSION

Vesicovaginal fistula most commonly occurs as a complication of gynaecological operations with an increasing trend of bladder injury with secondary fistulisation as laparoscopic surgery in pelvis is becoming more common.[1,2] Vesicovaginal fistula is a not a common complication of laparoscopic nephroureterectomy.[4,5] In our patient, a 7 cm Gibson incision was given for the ureterectomy as there was a struggle in taking out the cuff of the bladder and deep sutures were applied for the cuff closure. Therefore, it is possible some part of the vagina might have come into the suture and subsequently formed the fistula. Another possibility is the role of diathermy. This was applied only to excise the bladder cuff and it is unlikely to be responsible for the fistula. However, since the patient was obese and dissection was quite deep in the pelvis, it could have been a distant possibility. In conclusion, we recommend in an obese patient one should give a more liberal incision to have good exposure deep in the pelvis while closing the bladder cuff. One should also avoid taking deep bites with the suture, to avoid inadvertent injury or entrapment of the vagina in the closure. Careful closure of the bladder defect under good vision is mandatory to avoid such occurrence.
